# Singing for lung health following completion of pulmonary rehabilitation: feasibility of a randomised controlled trial

**DOI:** 10.1136/bmjresp-2025-003236

**Published:** 2026-01-06

**Authors:** Adam Lewis, Peter Jung, Parris Williams, Jennifer Steinmann, Karen A Ingram, Noah Longley, Puja Trivedi, Stuart Clarke, Helen Lammin, George Edwards, Maria Koulopoulou, Arun Sureshkumar, Anna Moore, Paul E Pfeffer, Leanne Reardon, Kim Sorley, Jarvis Kenman, Brendan DeLuca, Michelle Maguire, Laura-Jane Smith, Sarah Elkin, Adam Lound, Laura Moth, Penelope Rickman, Sharon Alexander, Natasha Lohan, Emily Garsin, Susan Young, Amanda Harris, Rosie Watters, Cleo Lane, Claire M Nolan, Joy Conway, William D-C Man, Winston Banya, Nana Anokye, Keir Elmslie James Philip, Phoene Cave, Nicholas S Hopkinson

**Affiliations:** 1 School of Health Sciences, University of Southampton, Southampton, UK; 2 College of Health, Medicine and Life Sciences, Department of Health Sciences, Brunel University London, London, UK; 3 Department of Health Sciences, Brunel University London, London, UK; 4 Guy's and St Thomas’ NHS Foundation Trust, London, UK; 5 National Heart and Lung Institute, Imperial College London, London, UK; 6 Royal Brompton and Harefield Hospitals, Guy's and St Thomas’ NHS Foundation Trust, London, UK; 7 King’s College Hospital NHS Foundation Trust, London, UK; 8 Barts Health NHS Trust, London, UK; 9 South London Clinical Research Network, London, UK; 10 Imperial College Healthcare NHS Trust, London, UK; 11 HCS Belfast Health and Social Care Trust, Belfast, UK; 12 The Musical Breath Ltd, Eastbourne, UK

**Keywords:** Pulmonary Rehabilitation, Asthma, Bronchiectasis, Emphysema, Perception of Asthma/Breathlessness

## Abstract

**Background:**

Pulmonary rehabilitation (PR) is a highly effective intervention for people with chronic respiratory disease; however, it is not known how best to sustain its benefits. Clinical trials are needed to establish if participation in singing for lung health (SLH) groups following PR will improve health-related quality of life, healthcare utilisation and exercise capacity compared with usual care. A feasibility study would help to guide development of these trials.

**Methods:**

In a multicentre, mixed-methods randomised controlled feasibility trial, PR participants at four sites were prescreened at baseline assessment. An SLH taster session was included routinely as part of the PR programmes. Eligible PR completers were invited to take part in the trial and randomised to usual care or a 12-week SLH course. Feasibility outcomes included recruitment rate, intervention compliance (at least 8/12 sessions) and health economic analysis. Interviews with participants and study personnel were undertaken and thematic analysis of the results was completed.

**Results:**

Between October 2022 and November 2023, 1311 patients were assessed to start PR, 838 completed. Of those completing, 243 were ineligible to take part (predominantly due to vaccination status and excluded diagnoses for PR referral), and 531 declined. 64 people (33 female, mean (SD) age 69 (12), 41 ethnically white, 33 with chronic obstructive pulmonary disease, 16 with asthma, 9 with interstitial lung disease, 6 with bronchiectasis) were recruited, with 30 (93.8%) SLH and 29 (90.6%) controls completing the study. 20 (62.5%) of the SLH group completed at least 8/12 SLH sessions. There was enthusiasm for a definitive trial from participants, clinicians and singing group leaders’ perspectives, based on positive experiences of trial involvement. Improvements to recruitment strategy, intervention structure, outcome measures and staffing were suggested.

**Conclusions:**

A definitive randomised controlled trial of SLH post-PR appears feasible, with acceptable uptake and completion rates.

**Trial registration number:**

ISRCTN11056049.

**WHAT IS ALREADY KNOWN ON THIS TOPIC**

- Singing for lung health (SLH) has previously been shown to improve health-related quality of life for people with chronic obstructive pulmonary disease. Pulmonary rehabilitation (PR) is a gold standard intervention, but it is not known whether SLH groups can be delivered as a maintenance programme after PR completion, or whether a randomised controlled trial (RCT) comparing this approach to usual care is feasible.

**WHAT THIS STUDY ADDS**

- It is feasible to conduct an RCT investigating the clinical and cost effectiveness of a 12-week SLH post-PR maintenance programme compared with usual care.

**HOW THIS STUDY MIGHT AFFECT RESEARCH, PRACTICE OR POLICY**

- This study will inform the design and delivery of a definitive RCT. The feasibility methodology used in this study can be applied to other creative health interventions which may be considered as maintenance options post-PR.

## Introduction

 Respiratory diseases are among the leading causes of morbidity and mortality globally and affect one in five people in the UK^1^ costing approximately £11 billion per year.^2^ Despite optimal treatment, many individuals remain disabled by physical impacts to health-related quality of life^3^ and social isolation.^4^ Pulmonary rehabilitation (PR) is a complex intervention consisting of group-based education and exercise, primarily aimed at people living with activity-limiting breathlessness. 6–8-week programmes of PR are well established with the highest quality of evidence, based on multiple randomised controlled trials (RCTs), systematic reviews and meta-analyses, showing patient improvements in breathlessness, quality of life and exercise tolerance.^5^ Individualised exercise programme plans should be provided to patients after PR completion.^6 7^ The benefits of PR decline over time.^8 9^ Evidence suggests that PR maintenance programmes can improve health status, functional exercise capacity, exacerbations and mortality, but further high-quality RCTs are needed.^8^,^11^ There is currently no evidence-based consensus on what the optimal choice of exercise content should be for PR maintenance.^11^ A perceived barrier by participants is the lack of being in a fun group environment,^12^ and home-based PR maintenance options have not fared as well compared with in-centre options.^13^ Additional approaches are needed to extend the benefits achieved in PR, especially ones which can build communities of social support for patients with chronic respiratory disease (CRD) who experience loneliness, social disengagement and isolation with negative consequences to their health.^14^

Singing for lung health (SLH) has the potential to be offered as a choice of PR maintenance activity. It is a creative health activity which addresses physical, mental and social needs for patients with respiratory disease.^15^,^18^ SLH groups are led by individuals who have received specific training to deliver group singing sessions for people with respiratory disease. If delivered after PR completion, SLH participation could address the need for a maintenance strategy at a critical point where patients have gained significant health benefit and a sense of belonging in a group environment.^19^ A recent study reported that PR completers who participated in singing as exercise during PR were more likely to report improved breathing control 4.7 years later, compared with those who performed resistance and endurance-based exercises within PR (PR standard exercises: 22%, SLH: 40%; p = 0.01).^20^

The aim of this study was to investigate the feasibility of conducting an RCT evaluating the impact of 12 weeks of SLH exercises compared with usual care for people with CRDs following completion of PR. We wanted to test components of a randomised controlled design to optimise a definitive clinical and cost-effectiveness study design.

## Methods

This study was prospectively registered (ISRCTN11056049) on 17 September 2021.

We conducted a parallel group, assessor-blind, randomised controlled feasibility study with a nested qualitative study to address the question: In people with chronic obstructive pulmonary disease (COPD), asthma, interstitial lung disease (ILD) or bronchiectasis who have completed PR, is an RCT comparing the effect of once weekly SLH to usual care alone feasible for participants to complete SLH Groups?

This paper has been written in accordance with the Consolidated Standards Of Reporting Trials (CONSORT) 2010 statement extension to pilot and feasibility studies.^21^

### Participant enrolment

Four PR centres following British Thoracic Society PR Quality Standards, who had SLH groups in their locality, took part in the study, with patients enrolled between October 2022 and November 2023. Following their baseline PR assessment, patients received a brief information leaflet describing the study. The PR programmes were also requested to include a SLH ‘taster’ session as part of the education programme. This included a brief PowerPoint introducing the trial and a demonstration of SLH exercises facilitated by the singing leader, which PR participants and clinicians were encouraged to join in with. Participants were provided with participant information sheets following this. Potential participants were invited to attend screening and assessment to take part in the clinical trial no later than a month after their discharge appointment from PR. The case report forms (CRFs), which were used during face-to-face research assessments, contained questions about participant demographics, medical history, recording of objective tests and questionnaire data. PR programmes were rolling PR programmes. All PR sites were in London, part of the London PR network, with clinicians working in teams with experience of research trials being performed in their Trust. PR groups also had SLH trained leaders who could work in each of the areas. A maximum of 15 participants per SLH group session was allowed according to venue risk assessments.

### Eligibility criteria

Participants were eligible for the study if they were at least 18 years old, had received three SARS-CoV-2 vaccines, an influenza and pneumococcus vaccine, clinically diagnosed with COPD, asthma, bronchiectasis or ILD, had stable respiratory health within the last 4 weeks (no exacerbations), completed at least eight sessions of PR and were able to provide informed consent. Participants were excluded if they had previously attended SLH group sessions, regularly participated in any other singing group activity, or if they had a life-limiting co-morbidity such as a terminal cancer diagnosis.

### Outcomes 

The study investigated a range of measures around recruitment and retention that might determine the feasibility of a definitive clinical trial. The primary feasibility outcome was completion of 12 weeks of SLH group classes. We hypothesised that 60% of individuals who were enrolled on the study after completing PR and randomised to the SLH arm would complete SLH (at least 8 out of 12 sessions). Other secondary feasibility, mechanistic and clinical outcomes were assessed by a face-to-face, blinded researcher assessment at 12 weeks and questionnaire survey at 24 weeks, including home exercise diary collection (feasibility), physical activity monitoring using McRoberts Dynaport Movemonitors (feasibility), adverse events (AEs) (collected by participant self-report, at a 6 weeks follow-up telephone call, 12-week follow-up and independent telephone contact made by participants) (clinical), number of control participants remaining in the study at 24 weeks, and qualitative interview data (feasibility and mechanistic). We also investigated the feasibility of collecting health economic data based on questionnaire data used in a previous physiotherapy RCT.^22^ Both study arms also received a phone call follow-up at 6 weeks to ask about home exercise activity.

Participants wore the multiaxial Dynaport Movemonitor around their waist following instructions given to the patient at their baseline assessment. This instruction was given to the patient verbally and printed out for them to take home. The device was charged fully for the participant before the baseline assessment and the charge lasted the whole week of use. The advice was to wear the Dynaport movemonitor continually for a week, apart from going into water, and it recorded step count and physical activity levels. A stamped addressed envelope was provided by the study team for the device return. The participants also completed the c-PPAC physical activity questionnaire developed by the PROactive consortium.^23^ The period of the activity monitoring was arranged so it preceded completion of the questionnaire (i.e so that the 1-week recall period of the questionnaire incorporated the period of monitoring).

An AE was defined as any untoward medical occurrence in a clinical study subject who was administered a treatment and which did not necessarily have a causal relationship with this treatment (i.e, any unfavourable or unintended change in the structure (signs), function (symptoms) or chemistry (lab data), including occurrences unrelated to that product/procedure/device).

A serious AE (SAE) was defined as an untoward occurrence that:

- Resulted in death.
- Was life-threatening (places the subject, in the view of the Investigator, at immediate risk of death).
- Required hospitalisation or prolongation of existing hospitalisation (hospitalisation is defined as an inpatient admission, regardless of length of stay; even if it is a precautionary measure for observation; including hospitalisation for an elective procedure, for a pre-existing condition).
- Resulted in persistent or significant disability or incapacity (substantial disruption of one’s ability to conduct normal life functions).
- Was otherwise considered medically significant by the investigator.

A list of secondary outcomes is below in box 1 which is written in chronological order, at which point during the study they were collected and categorised into type of outcome. Additional information regarding the timeline of outcome data collection is provided in the online supplemental table 14.

Box 1 **Study outcomes**

1. The number of eligible participants completing PR programmes (feasibility).
2. Rates of PR completer participants commencing the trial (feasibility).
3. The percentage of PR programmes that successfully implement the singing taster session within their PR (feasibility).
4. Recruitment rate per site in the RCT (feasibility).
5. The recording of home practice SLH sessions in patient diaries (feasibility).
6. Change in exercise capacity within and between groups according to the differences in Incremental Shuttle Walk Test between baseline and 12-week outcomes (clinical).
7. Changes in lung function parameters of inspiratory capacity, peak flow, forced expiratory volume in one second, forced vital capacity, within and between groups according to changes from baseline to 12 weeks (clinical).
8. Change in physical function according to the short physical performance battery score and physical activity according to activity monitor data (clinical).
9. Change in maximum phonation time from baseline to 12 weeks (mechanistic).
10. An economic evaluation. This was analysed from baseline data collected from the cost questionnaire and the questionnaire completed once again at 12 and 24 weeks (feasibility).
11. The feasibility of collecting disease-specific and general health-related quality of life data according to completion rates of the COPD Assessment/Asthma Quality of Life Questionnaire/Kings Brief ILD questionnaire/Bronchiectasis Health Questionnaire, Voice-related QOL questionnaire, EQ-5D-5L (Collected at baseline, 12 weeks and 24 weeks) (feasibility/clinical).
12. The number of patients in the control arm who continue in the trial to 24 weeks (clinical).
13. The perspectives of stakeholders across sites (PR leaders, singing leaders and patients), regarding experiences and the design of the Trial (feasibility/mechanistic).

COPD, chronic obstructive pulmonary disease; EQ-5D-5L, EuroQol 5-Dimension 5-Level Questionnaire; ILD, interstitial lung disease; PR, pulmonary rehabilitation; QOL, quality of life; RCT, randomised controlled trial; SLH, singing for lung health.

### Randomisation

Assessor blind randomisation was on a 1:1 basis, based on a REDCap software (V.12) random sequence generated by the Trial statistician with variable block sizes.

### Study arms

#### Usual care

All study participants were prescribed home exercise advice as a continuation of the clinical PR programme. This included resistance and endurance training, including exercises such as bicep curls, lateral shoulder raises, push-ups against the wall, squats, sit-to-stands and step-ups using the British Lung Foundation home exercise diary. Individuals were further advised to record their total minutes of outdoor walking each day.

#### Intervention

Additionally, participants randomised to the intervention arm took part in once weekly SLH group sessions for 12 weeks. Singing leaders were all trained by PC, who is a world-leading expert in SLH delivery. PC coordinated and supported singing leaders throughout the trial. Singing is a complex intervention, involving postural and breathing support and vocal technique. SLH differs from participation in more generic singing activities by its focus on improving breath control and posture in relation to respiratory disease. A typical 60 min class includes physical warm-ups, breathing exercises, vocal warm-ups, songs and a cool down/relaxation. Physical warm-ups use body mobilisation and simple exercises as well as using imaginative play, action songs and body percussion. Breathing exercises focus on optimal use of supporting musculature during inhalation and exhalation as well as systematically extending the outbreath. Vocal exercises include unvoiced and voiced fricatives introducing the additional resistance of both a semioccluded vocal tract and vocal fold closure moving from passive tidal breathing to a consciously voiced exhale. Finally, ‘Primal sounds’ such as Hey, Ho, Ha, etc., were introduced to engage vocal mechanism and support.^24^ Such SLH exercises have been used successfully in previous studies.^25^,^27^ Instructions were given to participants to perform daily 15–20 min practice exercise sessions at home using the ‘Singing for Breathing’ CD, given to the participants at the start of their participation in the singing group. The CDs were also available in digital format https://www.themusicalbreath.com/2021/05/13/singing-for-breathing-cd-downloads/ depending on participant preference. The frequency of practice sessions was recorded using home exercise diaries.

Both study arms received the SLH taster session within their PR programme, although potential participants did not have to attend that education session to be eligible for study entry. A SLH taster session within a PR education programme is not considered usual care.

Due to funding limitations at the feasibility study stage, clinicians in PR teams were not funded for their time during which the trial was recruiting patients from PR services.

### Patient and public involvement

PR and SA were patient and public involvement leads in the grant development process and continued to be integral to the study, participating in steering committee meetings throughout the trial, reviewing documents and supporting dissemination. They both live with CRD. PR has completed PR and SA has significant experience of SLH.

### Statistical analysis

64 participants is a suitable sample size for intervention group feasibility studies.^28^ If we identified 64 eligible subjects, we estimated a participation rate of 80% to within a 95% CI of ±10%. We, therefore, aimed to reach 50 completers of the trial. We were guided in the choice of the sample based on the recommendation of Richard Hooper’s, ‘Justifying sample size for a feasibility’ guidance provided by the Research Design Service London and the audit undertaken by Billingham *et al*^29^ who concluded that across feasibility and pilot studies, the median sample size per arm was 36 (range 10–300) for trials with a dichotomous endpoint and 30 (range 8–114) for trials with a continuous endpoint.

Exploratory analysis of change in outcomes between baseline and the 12-week endpoint was calculated and compared between intervention and study arms using independent two-tailed t-test using intention to treat analysis. Missing change was imputed at zero change. A per-protocol sensitivity analysis was performed including those in the intervention arm that adhered to the design (attending at least 8 out of 12 SLH sessions). Further sensitivity analysis was performed excluding disease groups with less than 10 participants to assess whether these contributed to greater variability in outcome. Subanalysis was performed in the intervention arm meeting the primary endpoint of adherence to test the change from baseline to 12 weeks using paired two-tailed t-test. The time within metabolic equivalent categories was expressed as a percentage of total time in sedentary, moderate and vigorously active categories. Energy expenditure has previously been validated for Dynaport activity monitors.^30^

Standardised effect sizes (d) for change in outcomes at 12-week endpoint between arms were calculated using G*Power. Sample size estimates were calculated to power a prospective trial design at 90% with a 1:1 ratio (alpha 0.05, 1-beta 0.9, two-tailed). Specific sample sizes for d>0.4 were calculated.

### Qualitative assessment

Semistructured interviews with 10 SLH group participants (completers and non-completers), 5 PR leads and 5 singing leaders were performed to explore their experiences of the trial. Interviews were performed via MS Teams/Zoom or by telephone, depending on participant choice. ALe performed the interviews and analysis. He is experienced in both qualitative research and PR. Interviews were transcribed verbatim by ALe. Considering the interviewer’s experience being viewed as valuable in the interpretation of findings, reflexive thematic analysis^31^ was performed. Thematic analysis stages included familiarisation with the data by reading and re-reading the transcripts, generating initial codes, collating codes into working themes and gathering data associated with those codes. Following this, themes were reviewed and refined. A thematic map was drawn and revised, final themes named and included in the writing of this report.^31 32^ Qualitative methodological issues were also factored into the analysis considering findings will be acted on in the design of a future definitive trial.^33^ The topic guides for respiratory participants, singing leader staff and PR staff are provided in the online supplemental appendices. The qualitative data aimed to provide a greater depth of understanding and context to quantitative outcomes collected in addition to novel insights into experiences of trial participation and perceptions on the study design. Supporting quotes aligned to the themes can be found in online supplemental table 12 ‘Quotes from participants’.

## Results

**Figure 1:**
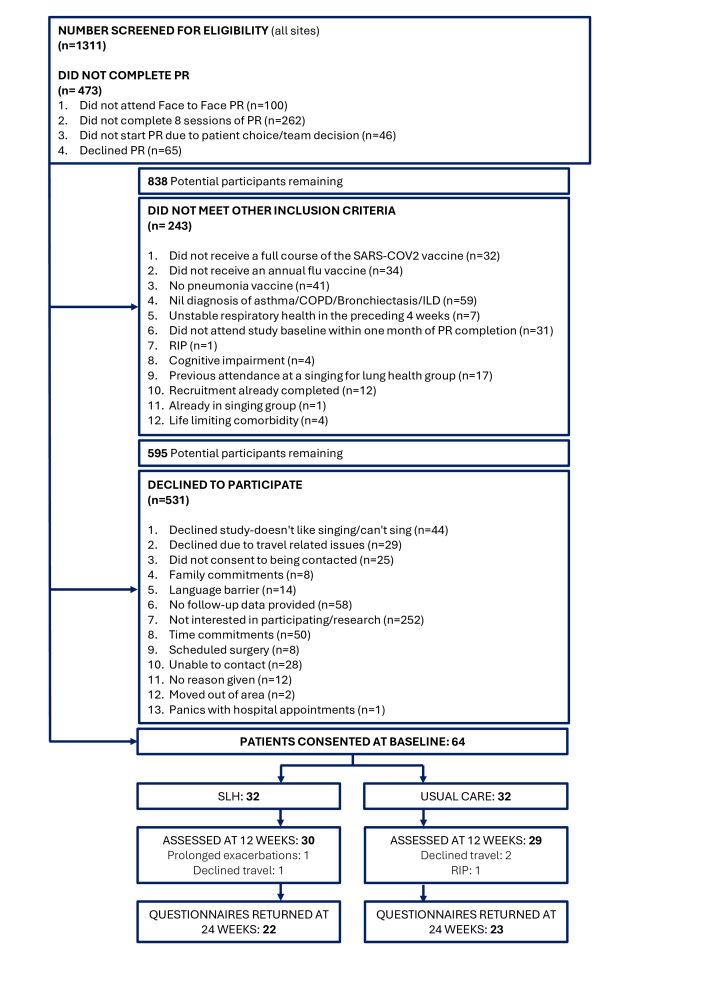
Participant recruitment screening and study completion flow chart. COPD, chronic obstructive pulmonary disease; ILD, interstitial lung disease; PR, pulmonary rehabilitation; SLH, singing for lung health.

1311 patients were screened for eligibility, of whom 595 were eligible. The main reason for ineligibility was non-completion of PR (n=473). 64 patients entered the trial, of whom 59 completed their follow-up 12-week reassessment. Further details on recruitment and study flow are in figure 1. Participants in the control and intervention groups were well matched at baseline, as seen in tables1  2.

**Table 1:** Baseline demographics

	Control n=32	SLH n=32
Diagnosis (%)		
Bronchiectasis	4 (13)	2 (6)
ILD	6 (19)	3 (9)
COPD	16 (50)	17 (53)
Asthma	6 (19)	10 (31)
Gender		
Female	16 (50)	17 (53)
Ethnicity (%)		
White	23 (72)	18 (56)
Non-white	9 (28)	14 (44)
Smoker (%)		
Never	12 (38)	7 (22)
Current	1 (3)	1 (3)
Ex	19 (59)	24 (75)
Age (yrs)	69.8 (13.1)	69.1 (11)
BMI (kg/m^2^)	26.6 (6.5)	29.2 (7.3)

Values are mean (SD) or n (%).

BMI, body mass index; COPD, chronic obstructive pulmonary disease; ILD, interstitial lung disease; SLH, singing for lung health.

**Table 2:** Patient outcome measures at baseline

	Control n=32	Singing for lung health n=32
Lung function		
FEV1/FVC	0.62 (0.19)	0.64 (0.18)
pFVC	79.69 (22.72)	82.63 (22.83)
pFEV1	62.99 (27.22)	67.27 (26.60)
IC	2.02 (0.64)	2.03 (0.57)
Respiratory rate	16.8 (4.8)	16.6 (3.8)
HRQOL		
EQ_vas	68 (18.2)	65.8 (18.6)
EQ_desc (IQR)	9.5 (7–13)	11 (9–15)
EQ_index	0.673 (0.208)	0.581 (0.255)
D12_score	10.2 (7.3)	12.5 (9.3)
VRQOL	83.7 (17.5)	84.1 (16.6)
MDP_A1	2.9 (2.2)	3.4 (2.7)
MDP_A2	2.5 (2.5)	2.7 (2.7)
MDP_SQ	2.4 (2.1)	2.8 (2.8)
KBILD (IQR)*	55.2 (45.2–64.4)	50.4 (49.1–53.5)
BHQ (IQR)*	47 (44–51)	50 (42–48)
CAT (IQR)*	22.5 (18.5–26)	19 (13–21)
AQLQ (IQR)*	5.97 (3.91–6.72)	4.21 (3.50–5.47)
Other		
Step count (IQR)	4557 (2586–6453)	2844 (1851–4802)
ISWT (m) (IQR)	310 (165–455)	310 (260)
SPPB (IQR)	12 (10–12)	12 (11–12)
EQ_desc (IQR)	9.5 (7–13)	11 (8–15)
MPTS	13.2 (6.1)	15.8 (6.9)
MET sed% (med)	90.40 (87.41–95.31)	92.73 (88.31)
MET mod% (med)	8.13 (3.47–11.05)	6.55 (3.66–9.74)
MET act% (med)	1.23 (0.22–2.21)	0.60 (0–1.86)
cPPAC total	65.0 (13.7)	63.8 (12.6)
cPPAC amount	62.9 (16.1)	61.5 (12.1)
cPPAC difficulty	67.2 (15.9)	66.2 (17.3)

Unless stated otherwise numbers are represented as mean (SD).

* Numbers of participants who completed the questionnaire matches the numbers of those participants with the disease specific to the questionnaire.

AQLQ, Asthma Quality of Life Questionnaire; BHQ, Bronchiectasis Health Questionnaire; CAT, COPD Assessment Test; COPD, chronic obstructive pulmonary disease; cPPAC, Clinical visit-PROactive Physical Activity in COPD; EQ_desc, Euroqol descriptive score; EQ_index, EQ Index score; EQ_VAS, EQ Visual Analogue Scale; FEV1/FVC, forced expiratory volume in 1 s/forced vital capacity; HRQOL, health related quality of life; IC, inspiratory capacity; ISWT, Incremental Shuttle Walk Test; KBILD, King’s Brief Interstitial Lung Disease questionnaire; MDP_A1, Multidimensional Dyspnoea Profile unpleasantness; MDP_A2, Multidimensional Dyspnoea Profile emotional response; MDP_SQ, Multidimensional dyspnoea profile sensory dimension; med, median; MET act%, percentage of time spent in METS≥3 METS with bout duration 10 min allowing interruption of 1 min; MET mod%, percentage of time spent in moderate activity (3–6 METS); MET sed%, percentage of time spent in sedentary activity (less than 3 METS); MPTS, maximum phonation time in seconds; pFEV1, percentage of FEV1; pFVC, percentage of predicted FVC; SPPB, Short Physical Performance Battery; VRQOL, voice-related quality-of-life score.

### Recruitment

The target of 64 participants was recruited to the study. 59 participants (92.2%) completed the study at the primary outcome timepoint of 12 weeks and 45 participants (70%) returned data at the 24-week follow-up. Five participants were recruited per month on average. Participants were assessed at their baseline study assessment on average 13.70 days (10.38 SD) after completing PR.

Participant consent per trial site ranged from 3 (2.6% eligible at site), 4 (4% eligible at site), 14 (8.7% eligible at site), to 43 (19% eligible at site).

### Primary outcome

20 out of 32 participants (62.5%) randomised to the SLH+home exercise arm completed 8 out of 12 sessions. Of those randomised, there were two non-starters of the singing groups; one participant was physically assaulted, injured and then unable to attend. The other participant reported it would be too much of a commitment following their initial assessment. There were 10 non-completers with reasons for drop out including: prolonged chest infection (four), pneumothorax (not related to intervention) (one), inappropriate location (two), travel disruption (storms) (one), caring responsibility (one), travelled abroad for bereavement (one).

23 (72%) of the control group returned postal questionnaire data at 24 weeks and 22 (69%) in the SLH group returned postal questionnaire data at 24 weeks. Therefore, there was not a greater drop-out in the control group in this feasibility study.

Exploratory comparative analysis of other clinical outcomes can be found in the online supplemental table 1 ‘Intention to treat analysis’, online supplemental table 2 ‘Per-protocol analysis’, online supplemental table 3 ‘Sensitivity analysis with Asthma and COPD cohorts only’.

Power calculations for outcome measures that could be used as a primary outcome in a definitive trial can be found in online supplemental table 4 ‘Power Calculations’.

Signals of efficacy from our feasibility trial were calculated by effect sizes (ES) and those reaching a threshold above 0.4 effect size for people with asthma and COPD include improvements in Short Physical Performance Battery (SPPB) (ES: 0.62), forced expiratory volume in first second (ES: 0.47), forced vital capacity (ES: 0.91), sedentary behaviour (ES: 0.43) and physical activity (ES: 0.76).

### SLH taster session integration into PR education programmes 

All PR centres integrated SLH taster sessions within their PR programmes.

### Physical activity monitoring data

Twenty-one participants in both groups (66% of 64) returned paired measurements of the Dynaport Movemonitor and 20 (62.5% of 32) in SLH group and 19 (59.4% of 32) in control group completed paired data for combined PROactive questionnaire and Dynaport MoveMonitor. Online supplemental table 5 ‘Physical activity monitoring’ shows pre to post 12-week changes in physical activity data.

### Home exercise diary completion

36/64 participants returned their home exercise diaries. However, of those returned, there was little consistency in the way these were completed. Many participants opted to record step counts even though they were advised to record total minutes walked daily.

### Health economic analysis

It was feasible to collect health economic data both by the collection of cost questionnaires at baseline (n=64/64), 12 weeks (n=59/64) and 24 weeks (45/64), and EQ5D5L data at each time point, although some participants found the cost questionnaire burdensome, particularly recording the number of visits to different healthcare professionals. The number of returns for the economic variables and descriptive statistics can be found in online supplemental table 6 ‘Baseline cohort qualifications’, online supplemental table 7 ‘Baseline health economics data’, online supplemental table 8 ‘12-week follow-up health economics data’, online supplemental table 9 ‘6-month health economics data’. Data were included of questions that were answered by at least 20 participants. The most popular health economic variable completed was EQ5D5L. 64, 59 and 44 returned completed EQ5D5L questionnaires at baseline, 12 weeks and 6 months, respectively.

### Adverse events

There were 53 AEs and 18 SAEs (one death) in the control group, and 50 AEs and 8 SAEs (0 deaths) in the SLH group. Respiratory SAEs included exacerbations/pneumonias requiring hospitalisation (8) and a pneumothorax (1). Non-respiratory SAEs related to falls (4), psychological (1), gastrointestinal (1), eyes, ears, nose, throat (1), dermatological (1), death (1), cancer (1), cardiovascular (4), musculoskeletal (1), genito-urinary (2). All SAEs were unrelated to the intervention. Total number of AEs per participant can be found in online supplemental table 10 ‘AEs’.

### Stakeholder perspectives

Twenty semistructured interviews were performed. Thirteen individuals with respiratory conditions were invited to participate in an interview. One participant had technical issues and later declined, one participant had worsening health and declined, and another participant was leaving the country and declined. Ten participants with respiratory conditions participated in interviews consisting of individuals living with different respiratory conditions, recruited from different PR centres, whether they were randomised to SLH or not and whether they completed a programme or not. Their demographics are in online supplemental table 11 ‘participant demographics for qualitative interviews’ in the appendices.

Five PR group leaders were interviewed, incorporating three out of four of the PR centres. Other clinicians were invited from the fourth centre but no response was received following an initial email and reminder. Five SLH group leaders were interviewed, representing all four singing group venues.

### Thematic analysis

Thematic analysis was focused on both understanding the feasibility of the trial design to make suitable adaptations for a future definitive study, whilst also understanding the meaning of SLH for participants in the context of PR.

**Figure 2:**
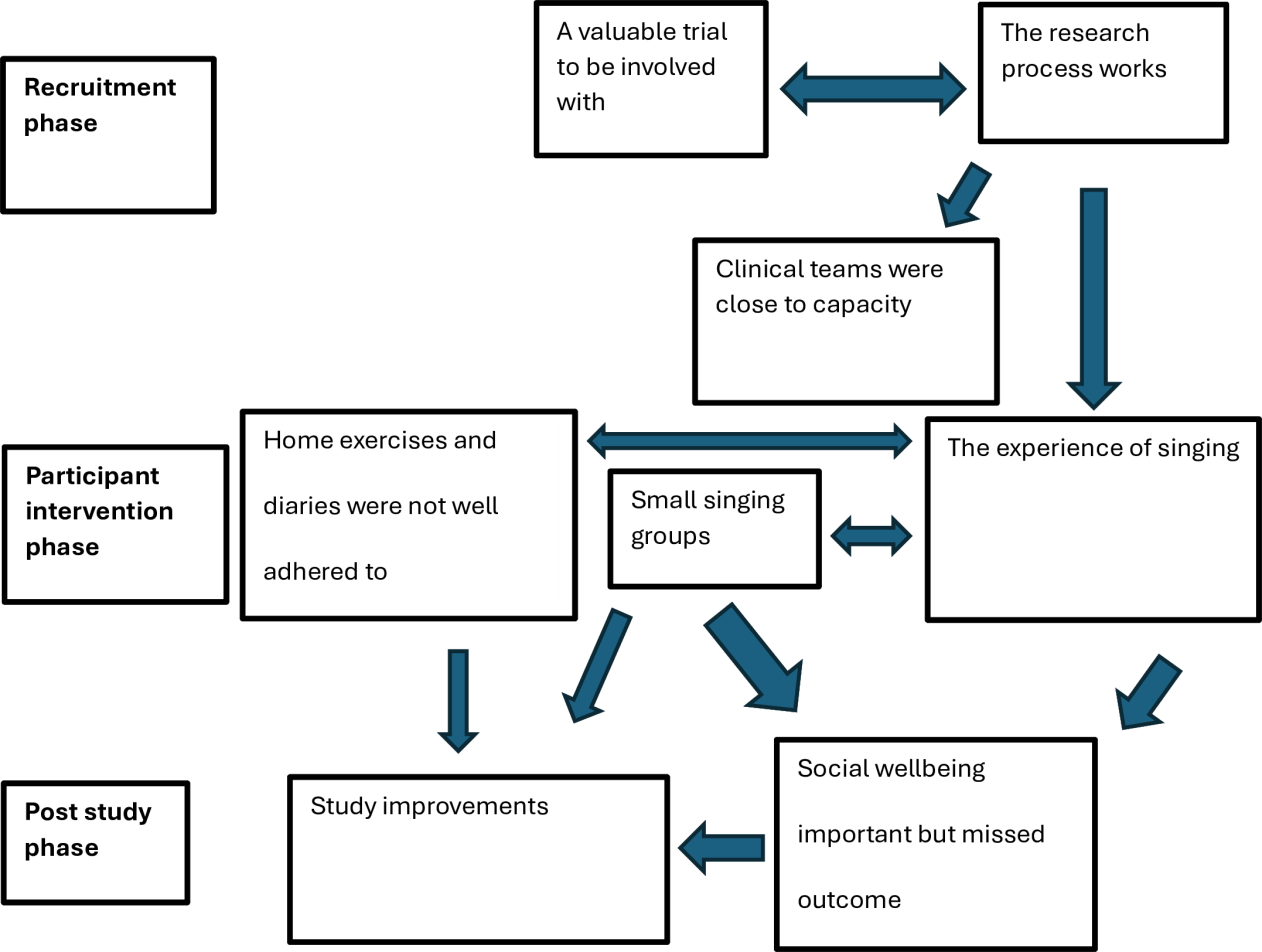
Thematic map.

Themes included: Clinical teams were close to capacity; A valuable trial to be involved with; The research process works; Small singing groups; Home exercises not well adhered to; The experience of singing; Social well-being an important but missed outcome; Suggested study improvements. Figure 2 shows the thematic map.

### Clinical teams were close to capacity

Multiple teams had staffing difficulties and high turnover during the trial period and were also focused on PR accreditation and other research studies. There was questionable ‘buy-in’ to the trial from some of the clinical teams with a perception from some singing leaders that there was some resistance to an alternative way of working. The study screening log was not always kept up to date, and in some sites, communication between the clinical team and singing leaders was suboptimal. On the other hand, other sites reported being able to integrate the research process into their practice well.

### A valuable trial to be involved with

Clinicians, patients and singing leaders all felt the trial was valuable. Being involved in research was a good learning opportunity for some clinical staff, and participants felt it was a privilege to be given the opportunity. It was an enjoyable study to be involved with and easy to understand from participant and clinician perspectives. Staff reported they would be keen to be involved in related future studies.

### The research process works.

Clinicians were able to discuss the study appropriately with potential participants during PR in a timely manner that did not overburden the PR assessment process. Overall, the study assessment made sense to participants, partly due to the overlap with known PR assessments. Participants reported they understood the importance of randomisation, and the timing of the study after PR was ideal with the singing dose being appropriate. The singing taster sessions were implemented, and they acted as a trigger to discuss the trial and an opportunity to challenge perceptions regarding the purpose of singing within the trial.

### Small singing groups

Singing groups were small across sites during the trial, due to the rolling nature of recruitment, the process of randomisation, recruitment rate and setting up four groups for the study. Participants were expecting to be in larger groups. Participants and singing leaders felt that although there was value in treating individuals in smaller groups or even on a one-to-one basis, there was huge benefit that came from the group support which was specific and different to that provided in a PR environment.

### Home exercises and diaries were not well adhered to

Prolonged physical activity maintenance post-PR was an acknowledged challenge from both participant and physiotherapist perspectives. Participants could not keep up with the volume of written tasks to do with home exercise diary completion, and the completion of diaries themselves was deliberately not enforced within the SLH sessions. Some felt they needed further instruction on the home exercises at assessment, but in contrast, other participants either chose different exercises that fit in with an existing app or had done similar exercises in the past, so knew what to do without the need for a home PR diary.

### The experience of singing

The experience of singing was discussed in terms of how it works, comparisons with PR, focus on breathing control often via the acronym SPLAT (Singers Please Lose Abdominal Tension), and the common belief of ‘I can’t sing’. Most participants who were interviewed and participated in sessions found benefits in their day-to-day lives, from enjoyment, feeling they were more motivated to do things, being in control of their breathing, or more comfortable with their condition. However, a participant also noted having to leave the class before the end of a session because she found it difficult, embarrassing and upsetting.

### Social well-being important but missed outcome

Participants thought the experience of SLH was inherently social and the environment set up to encourage social interaction, regarding leaving the house, socialising with others, gaining confidence and self-esteem. The benefits, however, were also described as intangible and not being able to determine a measurable difference from before the trial.

### Study improvements

Participants, singing leaders and clinicians all suggested study improvements. Participants thought the case report form was too complicated and not all questions were necessary or related to them. It was reported that participants in PR patient identification centre locations are reluctant to leave the local area and not keen to travel across London for assessments, and so local assessments with clinician researcher split roles would be advantageous. There were also suggestions made regarding home exercise diary and progression adaptations, with the use of websites, videos, smartwatches and apps needing to be considered.

Based on the analysis of both qualitative and quantitative data, and further discussions with the study steering group, a set of recommendations has been created when considering the design of a future definitive study, in online supplemental table 13 ‘Recommendations for future study’. These recommendations have been categorised into those of ‘Recruitment’, ‘Intervention’, ‘Patient outcomes’ and ‘Staffing’.

## Discussion

This is the first RCT exploring the feasibility of running face-to-face SLH groups after PR completion. Here we compared the provision of two PR maintenance interventions. One study arm received advice to perform home PR exercises only. The other study arm received SLH group sessions, home SLH exercise advice and was asked to perform home PR exercises. This is also the first study to recruit individuals with multiple different chronic respiratory diseases into face-to-face SLH groups. The primary feasibility outcome was achieved in that 20/32 individuals completed at least eight out of 12 sessions, above the feasibility threshold that we had set. As part of the protocol, participants were able to miss up to two sessions before returning to the group. However, if they missed a third session, they would then be deemed a non-completer, and other participants would be allowed to enter the group as part of a rolling programme. Travel and location issues were reasons for non-completion. Some SLH session locations had good parking facilities, but others were located closer to good public transport options (bus routes) with less parking. We did not provide transport for participants in this study. Those who had new caring responsibilities and bereavement had to move away from their home for a significant period and were therefore unable to continue to participate in the group classes. It was possible to recruit participants successfully within a month of completing PR, during which period people were maintaining benefit from PR.

Non-completion of at least eight sessions of PR was the reason for ineligibility for 20% of potential participants. Non-completion of PR is common in clinical practice. Another 100 people did not attend face-to-face PR. For those attending virtual PR, online SLH has been shown to improve quality of life for people living with COPD.^25^ Eligibility criteria were reviewed by a research ethics committee early in COVID-19 in the UK, advising the exclusion of participants without pneumococcal, influenza or COVID-19 vaccines which further reduced the numbers of those who could be eligible (107 out of 1311 (8%)). Patients can complete a group session of PR without these vaccines, and so it is possible these stringent exclusion criteria could be dropped for a future study.

Kaasgaard *et al*^34^ reported 295 eligible participants in their RCT comparing PR using standard exercise provision to PR with SLH as the exercise provision. However, they did not provide screening numbers. They recruited from 11 PR services compared with four services in our trial and had a drop-out rate of 28% post intervention, compared with 8% in our study. Philip *et al*^25^ performed an RCT of online SLH provision to standard care, and out of 391 screened, 121 consented to the trial. It could be expected that because the offer was an online intervention, there was improved uptake, because there was a reduced need for travel, particularly when this trial was conducted earlier in the COVID pandemic than our study. Reasons for decline in that study were similar to ours; participants had other commitments and were not interested in the research or intervention. 87% of participants were retained in the trial. Our trial also mirrors another study’s recruitment, comparing telerehabilitation to standard care.^35^ Hansen *et al*^35^ compared telerehabilitation to standard PR. Out of 1099 who were provisionally thought of as eligible for entry, 714 were not eligible due to reasons similar to our study, such as declining PR. In other PR maintenance studies, Ries *et al*^8^ stated there were 190 eligible participants to enter their study and Güell *et al*^9^ stated 143 participants were eligible. However, no data on screening numbers were included in these studies. Spencer *et al*^36^ state 59 participants were randomised out of 119 who completed PR. In summary, ineligibility and declining research participation are common in interventional studies relating to PR and maintenance, and there is likely a selection bias with these. Eldridge *et al*^37^ recommend in the CONSORT extension to randomised pilot and feasibility trials that participant flow diagrams should include numbers of participants screened prior to the assessment of eligibility. Unlike some of the other trials previously referenced. We provide these data, which are both important for feasibility analysis and future trial decisions regarding recruitment site prioritisation.

There was significant heterogeneity in the numbers of eligible participants who were recruited per site. One site recruited 19% of eligible patients. This site was where the principal investigator and chief investigator worked, is a flagship national PR centre (re-accredited) and clinicians were used to supporting research. Furthermore, all participants were assessed in this Trust. There were no principal investigators in the other sites, which were patient identification centres. Some qualitative data suggested that for some participants’ energy was consumed just getting to places, so performing multiple physical assessments after this may not have been attractive. A future study should be funded to have a principal investigator in each site with local participant research assessments.

The participant flow through the study from baseline randomisation to the 12-week primary outcome timepoint and 6-month follow-up indicates feasibility for a future definitive study considering low rates of attrition and no difference in rates of attrition between groups, indicating willingness of participants to be randomised to the control group. Improvements have been suggested to reduce attrition at the 6-month follow-up time point, such as offering a face-to-face review or clinician telephone follow-up for questionnaire completion.

The James Lind Alliance breathlessness top 10 research priority setting exercise priority number two is focused on how support for breathlessness can be tailored for those from different ethnic and social backgrounds.^38^ 23 out of 64 (36%) of participants recruited were of non-white ethnicity. We have also collected health economic data suggesting a range of education and occupation backgrounds in this sample, suggesting singing is an intervention of interest for diverse populations with respiratory disease, and one that can certainly be tailored. This is important as demographic variables such as these are not always collected in clinical trials.^39^

The completion and return of physical activity monitors at both baseline and paired follow-up points in both groups was suboptimal (21/32 in each group). This is similar to other data published in a home PR study regarding physical activity data attrition.^40^ Participants in our qualitative analysis recommended the use of smartwatches and/or apps to record activity in future studies. New monitoring devices would need to be reviewed regarding the accuracy of their recording. Small studies have shown validity and perceived usefulness of smartwatches for COPD self-management.^30 41^

Participants rarely completed the home diaries according to prescription, and interestingly, often entered step counts rather than the total minutes of walking activity per day as advised. This may reflect poor patient instruction, design of the diaries, or both. Other studies in the field have shown good adherence to home exercise diary completion.^42^ The method of recording home exercises needs optimising for a future definitive trial. It could be made a lot simpler, for example, by having a tick to indicate that exercises have been performed on a particular day, or leaving blank if not.^36^ Technology could be integrated to use technologies such as ‘SPACE for COPD’ or ‘myCOPD’ to record home exercise as these are both recommended for use by the National Institute for Health and Care Excellence (NICE).^43^

The SPPB is a composite assessment of gait speed, sit to stand function and standing balance. It has previously been shown to be responsive to change^44 45^ in PR and has an established minimal clinical important difference (MCID) range of 0.83–0.96.^44^ This could be a reasonable primary endpoint in a future study. Improving breathing control may improve balance, considering the diaphragm is a postural muscle. Therefore, the SPPB could provide data related to a potential mechanism of improvement, while also showing signs of clinical effectiveness of PR maintenance. Our SPPB data indicate an effect size of 0.62, with a between-group difference of 0.87 (95% CI −0.11 to 1.86, p: 0.079) in asthma and COPD participants, which achieves the MCID.

For a future trial, we would consider a range of social-related outcomes, including social isolation (Duke Social Support Index),^46^ disconnectedness (Social Disconnectedness eight-item scale)^47^ and loneliness (University of California Los Angeles (UCLA) loneliness scale).^48^ The final choice of social outcomes will likely be finalised by patient and public involvement and engagement (PPIE) and stakeholder groups in the development of future grant applications.

### Strengths and limitations

This study indicates multiple components of feasibility regarding the trial design. This is valuable information for a future definitive study. This study recruited individuals from a range of PR programmes in diverse areas of London, reflecting generalisability of clinical practice and the inclusion of future recruitment sites. Participants, singing leaders and clinicians all valued the research and recommended a future study following their involvement in this study. A range of participant experiences, alongside clinician and singing leaders’ experiences of the study, was obtained in qualitative data, which enabled the generation of themes relevant to multiple stakeholders for a future study. The completion rate is a strength indicating the study was conducted well and participants did not find it too burdensome to return at 12 weeks to a single assessment location.

Many patients needed to be screened to enter the study. Furthermore, during COVID-19, the clinical teams were both focused on working through a waiting list of patients, some focusing on PR accreditation, and all services had issues with staffing. The numbers screened need to be in context with the feasibility outcomes in this specific study. Although the numbers screened were high, future clinical effectiveness studies would commence screening at PR completion. Participants in both study arms received the SLH taster sessions within PR education programmes prior to consenting to the study. This may have caused a selection bias or potentially contaminated the usual care group, but was thought advantageous to enable people to understand what SLH involved beyond the provision of the participant information sheet. To mitigate the potential of contamination, we followed up those in the usual care group to find out if they joined a singing group over the course of their 12-week follow-up period, and none had. PR teams should be provided with funding to support recruitment in a future study, which should also be resourced with the support of a clinical trial network.

## Conclusions

We performed a randomised controlled feasibility study comparing SLH to usual care. The trial showed multiple areas of trial feasibility including recruitment and retention through the trial, pre-to-post 12-week outcome completion, and support from singing leaders, physiotherapists and patients for a future definitive RCT. Improvements are required regarding the need of a Principal Investigator in each recruitment site, that has good PR completion rates. The home exercise diaries and health economic questionnaires need to be further adapted, and offering a face-to-face or telephone appointment for 24-week follow-up questionnaire data could be considered. A definitive clinical and cost-effectiveness RCT of SLH after PR completion is now warranted.

## Supplementary material

10.1136/bmjresp-2025-003236 online supplemental file 1

## Data Availability

Data are available on reasonable request.
